# Associations of Influenza Illness During Pregnancy with Pregnancy Loss and Program-Defined Child Health Monitoring Indicator: A Matched Cohort Study

**DOI:** 10.3390/idr18040082

**Published:** 2026-08-05

**Authors:** Yuanyuan Zhang, Yan Shao, Suizan Zhou, Qianlan Wu, Ningning Du, Mingzhi Zhang, Qian Feng, Lin Bao, Yuanyuan Pang, Yayun Tan, Pengwei Cui, Jun Zhang, Haibing Yang, Suping Zhang, Liling Chen, Ying Song, W. William Schluter

**Affiliations:** 1 Suzhou Center for Disease Control and Prevention, 16 Guangqian Road, Suzhou 215131, China; zhangyuanyuan@szcdc.cn (Y.Z.); duningning@stu.njmu.edu.cn (N.D.); zhangmingzhi@suda.edu.cn (M.Z.); m17372146770@163.com (Q.F.); baolin@szcdc.cn (L.B.); pangyuanyuan@szcdc.cn (Y.P.); tanyayun@szcdc.cn (Y.T.); cuipengwei@szcdc.cn (P.C.); sz_zhangj@163.com (J.Z.); yanghaibing@szcdc.cn (H.Y.); spzhang@szcdc.cn (S.Z.); 2 Maternal and Child Health Care Institute, Suzhou 215031, China; shao_yan_76@163.com (Y.S.); wuqianlan520@163.com (Q.W.); 3 U.S. Centers for Disease Control and Prevention, Atlanta, GA 30329-4018, USA; kei5@cdc.gov (S.Z.); kei2@cdc.gov (Y.S.); wbs8@cdc.gov (W.W.S.); 4 School of Public Health, Nanjing Medical University, Nanjing 211166, China; 5 School of Public Health, Suzhou Medical College, Soochow University, Suzhou 215123, China

**Keywords:** influenza, pregnancy loss, fetal health, infant health, cohort study

## Abstract

Background/Objectives: This prospective cohort study assessed the impact of seasonal influenza during pregnancy on fetal and infant health outcomes. Methods: Pregnant women with laboratory-confirmed influenza were matched (1:4) by age and pregnancy loss history with women without influenza from annual cohorts in Suzhou, China, during 2015–2018. Participants underwent twice-weekly follow-up for influenza illness, and medical records were linked to ascertain outcomes. Multivariable regression models were used to estimate associations. Results: We included 441 pregnant women with influenza and 1764 without it. Four (0.9%) in the influenza group had late pregnancy loss, whereas one (0.1%) in the non-influenza group did. Influenza was associated with late pregnancy loss (adjusted hazard ratio [aHR] 31.1, 95% Confidence Interval [CI]: 1.3–756.8, *p* = 0.035). Four (0.9%) in the influenza group experienced early pregnancy loss, while five (0.3%) in the non-influenza group did. Influenza was not significantly associated with early pregnancy loss (aHR 2.1, 95% CI: 0.4–10.4, *p* = 0.374). By 8 months of age, infants born to 198 of the 441 mothers (44.9%) in the influenza group met the criteria for the program-defined child health monitoring indicator, compared with infants born to 650 of the 1764 mothers (36.8%) in the non-influenza group. Overweight/obesity was the largest contributor: 142 (32.2%) versus 470 (26.6%). Maternal influenza was associated with higher odds of meeting program-defined child health monitoring indicator in offspring (adjusted odds ratio [aOR] 1.4, 95% CI: 1.1–1.8, *p* = 0.006), with a significant contribution from overweight/obesity (aOR 1.3, 95% CI: 1.0–1.7, *p* = 0.045). Conclusions: In this cohort, influenza during pregnancy was associated with an increased risk of late pregnancy loss and program-defined child health monitoring indicator in offspring, particularly overweight/obesity. Because the observed association with pregnancy loss was based on a small number of events and had a wide confidence interval, it should be interpreted cautiously and confirmed in larger studies. These findings are consistent with current recommendations supporting influenza vaccination during pregnancy to protect maternal and infant health.

## 1. Introduction

During the influenza A(H1N1)pdm09 pandemic, a study from China found that pregnant women, who comprised less than 3% of the population, accounted for 15% of severe cases and 20% of deaths [1]. Severe maternal outcomes due to the pandemic, such as admissions to intensive care units and the need for mechanical ventilation, along with adverse fetal and neonatal outcomes, including fetal death, preterm birth and low birth weight have been documented [2,3]. During seasonal epidemics, influenza attacks at least 7% of the approximately 200 million pregnant women worldwide each year [4]. However, compared to the pandemic, the impact of seasonal influenza on pregnancy outcomes is less well understood. 

Seasonal influenza usually causes mild symptoms in pregnant women [5,6], but they are over three times more likely to be hospitalized compared to non-pregnant women [7]. Evidence on associations between seasonal influenza during pregnancy and fetal and neonatal outcomes has been inconsistent. While some studies have reported associations with pregnancy loss, preterm birth, or impaired offspring health, others have observed little or no association with birth outcomes such as preterm birth, low birth weight, or small for gestational age [8,9,10,11,12,13,14]. Interpretation of this literature is complicated by substantial differences in study design and exposure assessment. Many previous investigations included women hospitalized with severe influenza, pandemic influenza, influenza-like illness, acute respiratory illness, or ecological measures of influenza activity rather than laboratory-confirmed seasonal influenza infections in community-based populations. 

Furthermore, relatively few studies have evaluated health outcomes beyond birth. Existing evidence on longer-term offspring outcomes remains limited and primarily focused on neurodevelopmental disorders or childhood obesity [8,11,13,15], with little evidence from prospective cohorts of laboratory-confirmed seasonal influenza during pregnancy. Consequently, the potential longer-term effects of laboratory-confirmed seasonal influenza during pregnancy on offspring health remain incompletely understood. This study aimed to evaluate the associations between influenza during pregnancy and fetal and infant health outcomes by linking longitudinal active surveillance data on laboratory-confirmed influenza from annual cohorts of community-dwelling pregnant women with maternal and child healthcare medical records. 

## 2. Materials and Methods

### 2.1. Study Design and Population

This matched prospective cohort analysis used data from a subsample of the China Respiratory Illness Surveillance among Pregnant women (CRISP) study [16]. From 2015 to 2018, CRISP enrolled about 5000 pregnant women annually from antenatal care facilities in Suzhou, one of the most developed cities in China. CRISP cohorts included pregnant women living in Suzhou who planned to deliver there and excluded those seeking only non-routine antenatal care, such as for low progesterone or threatened miscarriage [5]. Thus, CRISP represents generally healthy pregnant women in the community. Although we aimed to include pregnant women in different trimesters, the first routine antenatal care typically occurs around 12 weeks of gestation. Before 2020, influenza vaccination was listed as contraindicated during pregnancy in the Chinese Pharmacopeia [17], so no CRISP participants received the vaccine.

This study was approved by the Institutional Review Board (IRB) of Jiangsu Provincial Center for Disease Control and Prevention (SL2015-B005-05). Informed consent was obtained from all individual participants included in the study.

### 2.2. Data Sources and Variables

#### 2.2.1. Active Surveillance on Influenza Illness

CRISP participants completed an enrollment interview that included questions about demographic characteristics, underlying medical conditions (any medical problem diagnosed by a doctor or other health care provider before pregnancy that lasted for at least six months such as diabetes, asthma, heart disease, or cancer) and health behaviors. Study nurses followed up with participants twice a week from enrollment through the early postpartum period (less than two weeks) to identify acute respiratory illness (ARI). ARI was defined as having at least one respiratory symptom (such as cough, sore throat, stuffy nose, chest pain, or difficulty breathing) along with at least one systemic symptom (such as feverishness, temperature ≥ 38 °C, chills, or headache), or at least two respiratory symptoms. For any reported ARI, a combined throat and nasal swab sample was collected by a study nurse. The procedure involved taking a throat swab from the tonsillar area using a sterile swab, placing it into a sterile viral transport media tube, and cutting off the applicator stick. A new sterile swab was then inserted into one nostril to collect a nasal sample, which was also placed into the same tube, and its applicator stick was cut off. The combined throat and nasal swab samples were tested for influenza virus subtype or lineage using reverse transcription–polymerase chain reaction (RT-PCR) [5]. 

#### 2.2.2. Data Linkage

We linked the CRISP influenza active surveillance dataset to the Suzhou Maternal and Child Health Information System, an integrated electronic medical record system [16], to obtain fetal and infant health outcomes. Adverse fetal outcomes included early pregnancy loss (spontaneous abortion before 13 weeks gestation), late spontaneous abortion (loss between 13 and 21 weeks gestation) and stillbirth (loss at 22 weeks gestation or later). Late spontaneous abortion and stillbirth were combined as late pregnancy loss in association analysis due to small numbers. Adverse infant health outcomes included preterm birth (live births delivered before 37 weeks of gestation), low birth weight (singleton 37–42 weeks gestation < 2500 g), small for gestational age (SGA) infants per Chinese growth curves, and program-defined child health monitoring indicator. A program-defined child health monitoring indicator was defined as meeting one or more monitoring criteria during the first 8 months of life defined by the Jiangsu Provincial Child Healthcare Monitoring Program, including nutritional developmental disorders, birth defects, neurodevelopmental disorders, or abnormalities of the five sensory organs. Nutritional disorders included overweight/obesity per child health program guidelines [18], defined as a body mass index (BMI) greater than two standard deviations above the age-sex specific WHO Growth Reference [19]. Other nutritional disorders were combined due to small numbers, including inadequate weight gain in the first month, iron-deficiency anemia, stunting, and Vitamin D deficiency rickets. Possible confounding factors were also abstracted including previous pregnancy loss, gestational diabetes, and pregnancy-induced hypertension as documented in the medical records. 

### 2.3. Identification of the Full Analytic Sample and the Matched Subsample

**Figure 1:**
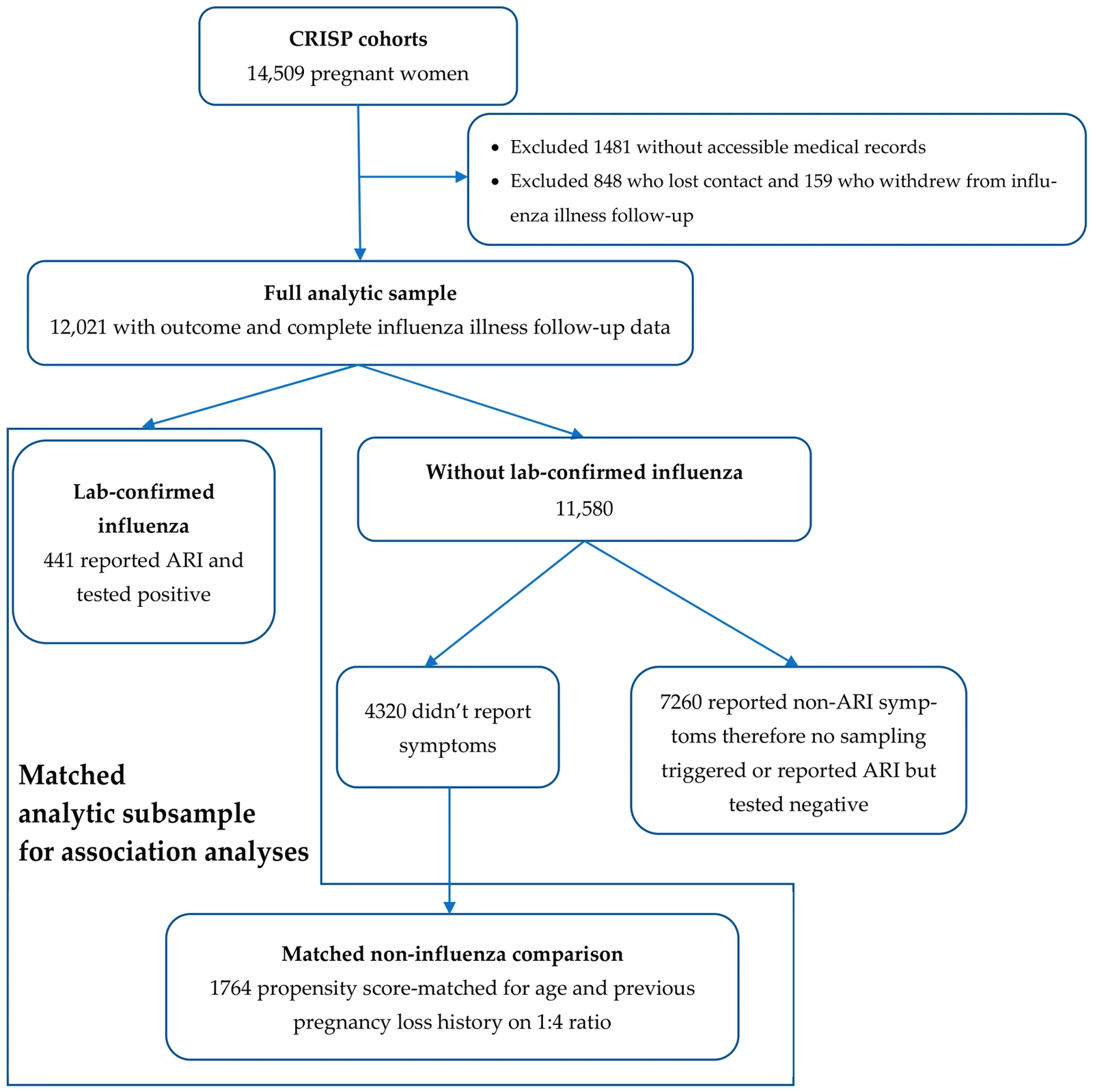
Study population. CRISP: China Respiratory Illness Surveillance among Pregnant women; ARI: acute respiratory illness.

The full analytic sample included CRISP participants with available medical records who completed active surveillance follow-up from enrollment until the end of pregnancy. Participants who reported ARI and tested positive for influenza were placed in the influenza group. Influenza severity was classified based on national clinical management guidelines [20]. Influenza illness without severe manifestations including consistent fever lasting more than three days, altered mental state, dehydration, pneumonia, worsening of underlying health conditions, or other complications were combined as mild-to-moderate illness category. The remaining participants, including those who reported ARI but tested negative and those who reported no symptoms or non-ARI symptoms and were not tested, were placed in the unmatched group without lab-confirmed influenza. From the asymptomatic participants, a matched non-influenza comparison group was selected at a 1:4 ratio using nearest-neighbor propensity score matching without replacement. Propensity scores were estimated using logistic regression with maternal age and previous pregnancy loss history as predictors (Figure 1).

### 2.4. Data Analysis

We described the characteristics of the full analytic sample, influenza group, unmatched group without lab-confirmed influenza, and matched non-influenza comparison group. Continuous variables were summarized using means or medians with standard deviations or interquartile ranges (IQR), while categorical variables were presented as frequencies and percentages. Variable categorizations were based on established clinical and routine practice standards. No imputation for missing data was performed because participants with incomplete influenza illness surveillance data were excluded, and outcomes data were obtained from local healthcare program, which incorporates routine quality-control procedures and provided essentially complete outcome ascertainment. Student’s *t*-test was used to compare the mean number of previous pregnancy losses per pregnancy between groups. Chi-square tests were used to compare categorical characteristics between groups, including age group, education, income, pre-pregnancy BMI, underlying conditions, reproductive history, smoking status, and pregnancy complications. To incorporate timing, Cox proportional hazards regression models were used to calculate the effect sizes of influenza during pregnancy on early pregnancy loss, late pregnancy loss, and preterm birth outcomes, reporting unadjusted and adjusted hazard ratios (HRs and aHRs) with 95% confidence intervals (CIs). The time scale of the Cox regression models was days since study enrollment, and participants were censored at the end of pregnancy. For other binary outcomes, logistic regression models were used to calculate unadjusted and adjusted odds ratios (ORs and aORs) and 95% CIs. Multivariable models (Cox or logistic) adjusted for educational attainment, annual household income, tobacco use, pre-pregnancy underlying diseases, pre-pregnancy BMI, parity, pregnancy-induced hypertension, gestational diabetes, and singleton versus multiple births based on prespecified epidemiological knowledge and biological plausibility. Eclampsia was not included because previous studies and our data showed no meaningful association with influenza risk, and the timing of eclampsia diagnosis in our participants did not support its confounding role in the relationship between influenza during pregnancy and the outcomes studied [21]. We also stratified influenza illness onset by trimester to evaluate any differences in outcomes based on the timing of influenza illness during gestation. The proportional hazards assumption for Cox regression was assessed using Schoenfeld residuals, and that multicollinearity and model fit were evaluated for logistic regression models. No major violations of model assumptions were identified. Statistical significance was defined as a two-tailed *p*-value of less than 0.05. All statistical analyses were conducted using R version 4.3.2.

## 3. Results

### 3.1. Study Population

Among the total 14,509 CRISP enrollees, 1481 (10%) were excluded due to the absence of medical records. Additionally, 848 (6%) who lost contact and 159 (1%) who voluntarily withdrew from the active influenza illness surveillance were excluded. This left a full analytic sample of 12,021 women, from which we identified 441 with RT-PCR-confirmed influenza illness and 1764 matched non-influenza comparison group for association analysis (Figure 1). Most participants in the full analytic sample were aged 20–34 years (91.0%), had a college education or higher (73.9%), maintained a pre-pregnancy BMI < 24 (84.4%), had no underlying chronic diseases (96.0%), and had never smoked (98.8%). All 441 influenza illnesses were classified as mild-to-moderate. Significant differences in parity and previous pregnancy loss per number of pregnancies were noted between the women with and without lab-confirmed influenza in the full analytic sample. These differences were no longer observed between the matched groups (Table 1).

### 3.2. Influenza and Pregnancy Loss

Four in 441 (0.9%) participants with lab-confirmed influenza had late pregnancy loss, compared to one in 1764 (0.1%) in the non-influenza group. The univariable regression model indicated that influenza during pregnancy was associated with late pregnancy loss (HR 17.6, 95% CI: 2.0–157.1, *p* = 0.010). This association remained significant in the multivariable regression model (aHR 31.1, 95% CI: 1.3–756.8, *p* = 0.035) (Table 2). 

Additionally, four in 441 (0.9%) participants with lab-confirmed influenza had early pregnancy loss, while five in 1764 (0.3%) in the non-influenza group did. The univariable regression model indicated that influenza during pregnancy was associated with early pregnancy loss (HR 3.7, 95% CI: 1.0–14.0, *p* = 0.049). However, this association was not confirmed in the multivariable analysis (aHR 2.1, 95% CI: 0.4–10.4, *p* = 0.374) (Table 2).

### 3.3. Influenza and Infant Health

Of the 441 mothers in the influenza group, 198 (44.9%) had infants who met the criteria for the program-defined child health monitoring indicator by the 8 months of age visit, compared with 650 of 1764 (36.8%) in the non-influenza group. In multivariable analysis, infants born to mothers with influenza were more likely to have program-defined child health monitoring indicator compared to the non-influenza group (aOR 1.4, 95% CI: 1.1–1.8, *p* = 0.006) (Table 2). 

By subcategory, infants born to 187 of the 441 mothers (42.4%) in the influenza group met the criteria for nutritional developmental disorders, compared with infants born to 619 of the 1764 mothers (35.1%) in the non-influenza group. Overweight/obesity was the largest contributor to nutritional disorders and to overall program-defined child health monitoring indicator, affecting infants born to 142 (32.2%) mothers versus 470 (26.6%) mothers in the two groups, respectively. Influenza during pregnancy was significantly associated with nutritional developmental disorders in live-born infants (aOR 1.4, 95% CI: 1.1–1.7, *p* = 0.004), including overweight/obesity (aOR 1.3; 95% CI: 1.0–1.7; *p* = 0.045) and other nutritional disorders (aOR 1.5; 95% CI: 1.0–2.2; *p* = 0.050). The association with neurodevelopmental disorders was not statistically significant (aOR 1.4, 95% CI: 0.6–3.3, *p* = 0.502). There were no statistically significant differences noted for preterm birth, low birth weight, or SGA infants (Table 2). 

Regarding the timing of influenza illness, the multivariable analysis suggested that infants born to mothers with influenza during the second trimester were more likely to have program-defined child health monitoring indicator compared to the non-influenza group (aOR 1.8, 95% CI: 1.2–2.5, *p* = 0.002). No statistical significance was found for influenza illness during the first trimester (aOR 1.4, 95% CI: 0.6–3.3, *p* = 0.429) or the third trimester (aOR 1.1, 95% CI: 0.8–1.6, *p* = 0.433) in relation to the risk of live-born infants being identified with program-defined child health monitoring indicator. Additionally, no statistically significant associations were found for other infant health outcomes based on the timing of influenza illness (Table 2). 

## 4. Discussion

This study found that pregnant women with mild-to-moderate influenza had a higher risk of late pregnancy loss compared to those without influenza. Additionally, infants born to mothers with influenza were more likely to meet the program-defined child health monitoring indicator during the first year after birth, with the contribution primarily from overweight/obesity. 

Our findings are consistent with previous evidence suggesting that mild-to-moderate influenza during pregnancy may increase the risk of late pregnancy loss. Multi-center cohorts of pregnant women in India, Peru, and Thailand found that those with influenza had a 10.7-fold higher risk of late pregnancy loss and their babies weighed an average of 55.3 g less at birth compared to those without influenza, with no differences in preterm birth, SGA or low birth weight [22]. An earlier review also indicated that influenza during pregnancy significantly raised the risk of stillbirth (3.62-fold) [14]. Our study found a similar link between influenza during pregnancy and late pregnancy loss. In addition, the univariable analysis suggested a possible connection to early pregnancy loss, although this was not confirmed in the multivariable analysis. However, a modeling study did suggest a link between influenza during pregnancy and early fetal loss [23]. This discrepancy may arise because many miscarriages occur in the first few weeks of pregnancy and often do not present at antenatal care facilities where our cohorts were enrolled. Therefore, our study may have missed the full impact of influenza on early pregnancy loss.

We observed a substantial proportion of infants classified with program-defined child health monitoring indicator in both the influenza and non-influenza groups. This is largely driven by the inclusion of overweight/obesity in the monitored child health indicator. Since 2015, the health program in the study site province has included infant overweight/obesity as a monitored child health indicator to enable early identification and intervention in response to rising overweight/obesity among children [19]. In addition, overweight/obesity plus the other sub-indicators were all measured cumulatively during the first 8 months of life in our study, rather than as a single cross-sectional proportion at one time point. It recorded whether an infant ever exceeded the threshold during this period. Because infant growth is dynamic, some infants may briefly exceed the threshold (for example, during rapid catch-up growth or short-term feeding patterns) and subsequently return to the expected range after routine counseling or adjustment. A cumulative measure captures these transient episodes, which increases the proportion compared with a cross-sectional measure at a single age. The high cumulative frequency of overweight/obesity does not imply persistent overweight or obesity at exactly 8 months. Instead, it reflects the need for follow-up, referral, confirmation and targeted intervention within the public health system. The program-defined child health monitoring indicator should be interpreted as a public health surveillance measure used within the local Child Healthcare Monitoring Program to identify infants who may benefit from further assessment or follow-up, rather than as an internationally validated clinical endpoint. The components and operational definition of this indicator may differ from those of similar measures used in other countries. Therefore, caution is warranted when generalizing these results to other healthcare settings.

The absence of statistically significant associations with preterm birth, low birth weight, and small-for-gestational-age infants in our observation should be interpreted cautiously rather than as evidence of no association. Our cohort consisted predominantly of healthy pregnant women with mild-to-moderate laboratory-confirmed influenza, which may have reduced our ability to detect modest associations compared with studies including severe or hospitalized influenza cases. In addition, the relatively low frequency of these outcomes may have limited statistical power to detect modest associations. Differences in study populations, influenza severity, and exposure assessment may also contribute to the heterogeneous findings reported in the literature. Small studies, particularly those looking at mild-to-moderate influenza illnesses, have shown inconsistent results regarding the relationship between influenza during pregnancy and newborn health, especially regarding short-term outcomes at delivery [10,12,14,22]. However, meta-analyses and larger studies have found moderate associations between influenza during pregnancy and various newborn outcomes. A recent meta-analysis of 24 studies involving about 25 million patients showed that influenza during pregnancy was associated with 1.52 times higher risk of preterm birth, although there was high variability among the studies (*I*^2^ = 97.4) [12]. In the U.S., a study of 0.3 million births found that an interquartile range increase in a composite measure of influenza-like illness activity and percent test-positive of influenza in the community were linked to a 1.014-fold increase in preterm birth during the same week [9]. Another study in New Zealand involving 0.8 million pregnancies from 2003 to 2018 showed that pregnant women hospitalized for ARI during influenza season had a 1.64-fold greater risk of low birth weight and a 1.50-fold higher risk of preterm birth compared to those pregnant women hospitalized for non-ARI [24]. This study provided additional evidence suggesting that influenza during pregnancy may have a moderate impact on offspring health beyond delivery, including an increased risk of overweight and obesity by eight months postpartum. Similar moderate longer-term effects on infant health have also been reported elsewhere [8]. For instance, national data from Korea indicated that children born to mothers with influenza had higher rates of being overweight 30–80 months after birth [15]. 

Several biological mechanisms have been proposed to explain how maternal influenza infection may influence fetal and infant health. Influenza infection during pregnancy may affect the maternal–fetal interface through placental inflammation or maternal immune activation, potentially disrupting placental function and fetal development [25,26,27]. Maternal systemic inflammatory responses and cytokine production have also been proposed as indirect pathways through which influenza infection could contribute to adverse pregnancy outcomes, including pregnancy loss [26,27]. In addition to pregnancy outcomes, maternal infection may influence fetal metabolic and immune development. Experimental and epidemiologic studies have suggested that inflammatory responses during pregnancy could alter fetal metabolic programming, providing one possible explanation for the modest association with infant overweight/obesity observed in our study [27,28,29]. However, these biological pathways remain incompletely understood, and our observational data cannot establish the underlying mechanisms. Future mechanistic studies are warranted.

Previous studies have also reported associations between maternal influenza or other prenatal infections and longer-term neurodevelopmental outcomes, including seizures and neurodevelopmental disorders [8,11,13,30]. Because our study followed children only through 8 months of age and did not assess these outcomes, our findings should not be interpreted as evidence supporting or refuting those associations. Further longitudinal studies are needed to determine whether laboratory-confirmed seasonal influenza during pregnancy is associated with longer-term child health outcomes. Furthermore, because this study included predominantly healthy, unvaccinated pregnant women from a single developed city in China, the generalizability of the findings to other populations, healthcare systems, or settings with routine maternal influenza vaccination may be limited. We selected matched controls from participants who did not report any symptoms during follow-up to minimize potential exposure misclassification and provide a comparison with pregnancies without evidence of respiratory illness. This design does not distinguish the effects of influenza from those of other respiratory illnesses. As an observational study, our findings demonstrate associations rather than causal relationships. Although we adjusted for measured confounding factors, residual confounding from unmeasured or incompletely measured variables cannot be excluded. 

This study has several limitations. First, the study population may have been subject to selection bias arising from missing medical records, loss to follow-up, and exclusion of women who enrolled after early pregnancy. We could not enroll pregnant women earlier as most first antenatal visits typically occurred around 12 weeks of gestation. Since most pregnancy losses happen in the initial weeks, our data on early pregnancy loss may be incomplete, indicating a need for future research. Additionally, we excluded pregnant women seeking care for low progesterone or threatened miscarriage, as we could not ascertain their influenza infection status prior to enrollment. Consequently, the participants represented healthy pregnancies in a well-developed city with strong maternal and child healthcare services. This may have resulted in a lower prevalence of risk factors and adverse outcomes compared to the general pregnant population. The rarity of pregnancy loss may reduce the stability of multivariable estimates. Furthermore, the sample size was inadequate to narrow the confidence intervals for rare outcomes, such as late pregnancy loss in healthy participants. The observed association should be interpreted cautiously because of the rarity of pregnancy loss events, the wide confidence interval, and the potential for immortal-time bias arising from modeling influenza as a fixed exposure. Larger studies are needed to confirm these findings. This study also lacked power to detect associations with specific nutritional disorders beyond overweight/obesity, and with specific neurodevelopmental conditions. Future research could quantify the relationships between influenza during pregnancy and these specific components of program-defined child health monitoring indicator. Because our database records whether a child met the monitoring threshold at any time during follow-up, rather than whether the condition persisted, we were unable to distinguish persistent from transient abnormalities. Future studies with repeated longitudinal anthropometric measurements are needed to evaluate the persistence of these findings.

## 5. Conclusions

In conclusion, influenza illness during pregnancy was associated with late pregnancy loss and an increased likelihood of meeting the program-defined child health monitoring indicator in offspring, driven primarily by infant overweight/obesity in the study cohort. Larger studies are necessary to further quantify the impact of influenza on early pregnancy loss and other specific types of nutritional disorders and neurodevelopmental disorders. These findings are consistent with current recommendations supporting influenza vaccination during pregnancy to protect maternal and offspring health.

## Figures and Tables

**Table 1:** Characteristics of participants.

Characteristics	Full Analytic Sample (*N* = 12,021)	Influenza (*N* = 441) A(H1N1)pdm09 (117) A(H3N2) (189) B Victoria (37) B Yamagata (98)	Without Lab-Confirmed Influenza (*N* = 11,580)	*p*-Value	Matched Non-Influenza Comparison ^a^ (*N* = 1764)	*p*-Value
Pre-pregnancy						
Median age, years (interquartile range)	28 (26–31)					
Age group, year-old						
<20	48 (0.4)	0 (0)	48 (0.4)	0.268	2 (0.1)	0.695
20–34	10,937 (91.0)	408 (92.5)	10,529 (90.9)		1618 (91.7)	
35–49	1036 (8.6)	33 (7.5)	1003 (8.7)		144 (8.2)	
Education						
9th grade or lower	853 (7.1)	35 (7.9)	818 (7.1)	0.315	81 (4.6)	<0.001
10–12th grade	2230 (18.6)	92 (20.9)	2138 (18.5)		270 (15.3)	
College or higher	8887 (73.9)	313 (71.0)	8574 (74.0)		1410 (79.9)	
Annual household income, Chinese currency						
<200,000	9492 (79.0)	341 (77.3)	9151 (79.0)	0.828	1424 (80.7)	0.337
≥200,000	1760 (14.6)	61 (13.8)	1699 (14.7)		299 (17.0)	
Pre-pregnancy body mass index, kg/m^2^						
<18.5	1889 (15.7)	66 (15.0)	1823 (15.7)	0.959	275 (15.6)	0.959
18.5 to <24	8260 (68.7)	305 (69.2)	7955 (68.7)		1202 (68.1)	
24 to <28	1513 (12.6)	56 (12.7)	1457 (12.6)		235 (13.3)	
≥28	348 (2.9)	14 (3.2)	334 (2.9)		52 (2.9)	
Underlying chronic disease ^b^						
No	11,540 (96.0)	416 (94.3)	11,124 (96.1)	0.056	1703 (96.5)	0.039
Yes	473 (3.9)	25 (5.7)	448 (3.9)		61 (3.5)	
Previous pregnancy loss						
No	6538 (54.4)	223 (50.6)	6315 (54.5)	0.108	892 (50.6)	1.000
Yes	5483 (45.6)	218 (49.4)	5265 (45.5)		872 (49.4)	
Parity						
1	5083 (42.3)	161 (36.5)	4922 (42.5)	0.037	694 (39.3)	0.453
2	3840 (31.9)	151 (34.2)	3689 (31.9)		599 (34.0)	
3 or more	3090 (25.7)	129 (29.3)	2961 (25.6)		471 (26.7)	
Previous pregnancy loss per number of pregnancies (mean, standard deviation)	0.291 (0.317)	0.277 (0.294)	0.291 (0.317)	0.002	0.268 (0.285)	0.383
Current pregnancy						
Have ever smoked						
Yes	69 (0.6)	2 (0.5)	67 (0.6)	1.000	16 (0.9)	0.553
No	11,880 (98.8)	438 (99.3)	11,442 (99.4)		1744 (98.9)	
Pregnancy-induced hypertension						
Yes	157 (1.3)	4 (0.9)	153 (1.4)	0.668	23 (1.3)	0.633
No	11,417 (95.0)	428 (97.1)	10,989 (94.9)		1741 (98.7)	
Gestational diabetes						
Yes	1277 (10.6)	41 (9.3)	1236 (10.7)	0.347	176 (10.0)	0.721
No	10,298 (85.7)	391 (88.7)	9907 (85.6)		1588 (90.0)	
Eclampsia in pregnancy						
Yes	101 (0.8)	5 (1.1)	96 (0.8)	0.430	14 (0.8)	0.562
No	11,473 (95.4)	427 (96.8)	11,046 (95.4)		1750 (99.2)	
Singleton						
No	145 (1.2)	6 (1.4)	139 (1.2)	0.826	32 (1.8)	0.682
Yes	11,104 (92.4)	427 (96.8)	10,677 (92.2)		1726 (97.8)	

Data are *n* (%), otherwise as indicated. ^a^ Propensity score-matching factors include age and loss of pregnancy history. ^b^ Underlying chronic disease refers to any medical problem diagnosed by a doctor or other health care provider before pregnancy that lasted for at least six months such as diabetes, asthma, heart disease, or cancer.

**Table 2:** Lab-confirmed influenza illness and fetal and infant health outcome.

			Univariable Regression Model		Multivariable Regression Model ^a^	
	Influenza ^b^ (*N* = 441)	Matched Non-Influenza Comparison (*N* = 1764)	Effect Size (95% CI) ^c^	*p*-Value ^d^	Effect Size (95% CI) ^c^	*p*-Value ^d^
Fetal health						
Early pregnancy loss	4 (0.9)	5 (0.3)	3.7 (1.0–14.0)	0.049	2.1 (0.4–10.4)	0.374
Late pregnancy loss	4 (0.9)	1 (0.1)	17.6 (2.0–157.1)	0.010	31.1 (1.3–756.8)	0.035
Infant health						
Preterm birth	18 (4.1)	94 (5.3)	0.7 (0.4–1.1)	0.141	0.7 (0.4–1.2)	0.186
	Early trimester		0.6 (0.2–2.6)	0.513	0.7 (0.2–3.0)	0.662
	Second trimester		0.4 (0.2–1.1)	0.072	0.4 (0.2–1.0)	0.059
	Third trimester		1.0 (0.5–1.8)	0.878	1.0 (0.5–1.8)	0.944
Full-term low birth weight	11 (2.5)	49 (2.8)	1.0 (0.5–2.0)	0.902	0.9 (0.4–1.9)	0.795
	Early trimester		2.4 (0.5–10.8)	0.263	1.2 (0.1–10.0)	0.866
	Second trimester		0.7 (0.2–2.4)	0.579	0.8 (0.2–2.6)	0.672
	Third trimester		1.1 (0.5–2.6)	0.845	1.0 (0.4–2.5)	0.951
Small for gestational age	32 (7.3)	156 (8.8)	1.0 (0.7–1.5)	0.956	0.9 (0.6–1.4)	0.595
	Early trimester		1.2 (0.3–4.2)	0.789	0.8 (0.2–3.7)	0.771
	Second trimester		0.6 (0.3–1.2)	0.149	0.6 (0.3–1.4)	0.216
	Third trimester		1.3 (0.8–2.2)	0.259	1.1 (0.6–1.9)	0.778
Program-defined child health monitoring indicator ^e^	198 (44.9)	650 (36.8)	1.4 (1.1–1.8)	0.002	1.4 (1.1–1.8)	0.006
	Early trimester		1.5 (0.7–3.1)	0.312	1.4 (0.6–3.3)	0.429
	Second trimester		1.7 (1.2–2.4)	0.002	1.8 (1.2–2.5)	0.002
	Third trimester		1.3 (0.9–1.7)	0.151	1.1 (0.8–1.6)	0.433
Nutritional developmental disorders	187 (42.4)	619 (35.1)	1.4 (1.1–1.7)	0.004	1.4 (1.1–1.7)	0.004
Overweight/obesity	142 (32.2)	470 (26.6)	1.3 (1.1–1.8)	0.009	1.3 (1.0–1.7)	0.045
Other nutritional disorders	45 (10.2)	149 (8.4)	1.4 (1.0–2.0)	0.079	1.5 (1.0–2.2)	0.050
Birth defects	1 (0.2)	6 (0.3)	NA	NA	NA	NA
Neurodevelopmental disorders	7 (1.6)	20 (1.1)	1.4 (0.6–3.3)	0.441	1.4 (0.6–3.3)	0.502
Abnormalities of the five sensory organs	0 (0.0)	1 (0.1)	NA	NA	NA	NA

Data are *n* (%) otherwise as indicated. NA: not applicable due to small sample size. CI: confidence interval. Percentages are based on the total number (*N*) of enrolled mothers in each group. Model estimates were calculated among participants eligible for the specified outcome. ^a^ Multivariable regression models adjusted the level of educational attainment, annual household income, tobacco use, pre-pregnancy body mass index, underlying diseases, parity, pregnancy-induced hypertension, gestational diabetes, and singleton versus multiple births. ^b^ To assess the average association with infant health, we compared outcomes between the influenza group, which included all instances of influenza occurring during different trimesters of pregnancy, and the non-influenza group. Additionally, we conducted separate comparisons of infant health outcomes for influenza illnesses that developed in the early, second, and third trimester against the outcomes of the non-influenza group. ^c^ To incorporate timing, Cox proportional hazards regression models were used to calculate the effect sizes of influenza during pregnancy on early pregnancy loss, late pregnancy loss, and preterm birth outcomes, reporting unadjusted and adjusted hazard ratios with 95% CIs. For other binary outcomes, logistic regression models were used to calculate the effect sizes, reporting unadjusted and adjusted odds ratios and 95% CIs. ^d^ *p*-values refer to the comparisons of influenza to matched non-influenza. ^e^ A program-defined child health monitoring indicator was defined as meeting one or more monitoring criteria during the first 8 months of life, including nutritional developmental disorders, birth defects, neurodevelopmental disorders, or abnormalities of the five sensory organs. Nutritional disorders included overweight/obesity per child health program guidelines, defined as a body mass index (BMI) greater than two standard deviations above the age-sex specific WHO Growth Reference. Other nutritional disorders were combined due to small numbers and included inadequate weight gain in the first month, iron-deficiency anemia, stunting, and Vitamin D deficiency rickets.

## Data Availability

The data that support the findings of this study are available from the corresponding author upon reasonable request.
